# Life is better but not without challenges: experiences following discharge from community-based residential mental health rehabilitation–a qualitative content analysis

**DOI:** 10.1007/s00127-024-02716-z

**Published:** 2024-07-16

**Authors:** Stephen Parker, Maddison Chapman, Marianne Wyder, Matthew Pommeranz, Rebecca Walgers, Frances Dark, Carla Meurk

**Affiliations:** 1 Metro South Addiction and Mental Health Service, Woolloongabba, QLD Australia; 2 Faculty of Medicine, University of Queensland, Herston, QLD Australia; 3 Metro North Mental Health, The Prince Charles Hospital, Chermside, QLD Australia; 4 Queensland Centre for Mental Health Research, University of Queensland, Wacol, QLD Australia; 5 Metro North Mental Health, Royal Brisbane & Womens Hospital, Herston, QLD 4006 Australia

**Keywords:** Psychiatric rehabilitation, Residential services, Consumer experiences, Qualitative research

## Abstract

**Purpose:**

Community-based residential mental health rehabilitation units for people experiencing severe and persistent mental illness are increasingly available in Australia. Research completed 20 years ago suggested that people leaving these services often experienced impoverished social lives and other challenges in the community. It is unclear whether contemporary consumers experience similar difficulties. This qualitative study explored contemporary consumers’ experiences after leaving community-based residential services.

**Methods:**

An inductive qualitative content analysis of individual interviews was completed with consumers 12–18 months following discharge from three community care units (CCUs) in Queensland, Australia. The interview schedule explored three questions: (1) What does life look like after leaving the CCU, (2) Has the CCU impacted their life, and (3) How could the CCU experience be improved? A convenience sample was used, with sampling continuing until thematic saturation was achieved. A member of the research team who had relevant lived experience actively supported the analysis and interpretation.

**Results:**

Seventeen interviews were completed. Three themes were identified: ‘life is better but not without challenges’, ‘the CCU helps you get ready to go out into the world’, and ‘strict rules are important but rigid expectations can be hard; things could be better’.

**Conclusion:**

Consumers reflected positively on their lives post-discharge from a community-based residential rehabilitation unit and viewed the service as having supported improvements in their lives. The findings suggest the appropriateness of optimism about the possibility of sustained improvements in quality of life after leaving community-based transitional residential rehabilitation support.

**Supplementary Information:**

The online version contains supplementary material available at 10.1007/s00127-024-02716-z.

## Introduction

There has been considerable expansion in publicly funded community-based residential mental health rehabilitation service capacity in Australia over the past two decades [1]. This trend contrasts with the shift from transitional residential treatment services to ‘choose-get-keep’ permanent housing initiatives like Housing First in countries such as the United States of America and Canada [2–4]. Some authors have argued that transitional residential treatment services are inappropriate and that permanent housing solutions are preferable [5, 6]. However, research considering optimal approaches to meet the accommodation and support needs of people affected by severe and persisting mental illness (SPMI) in the community remains limited [7, 8].

The Community Care Unit (CCU) model is the most common community-based residential mental health rehabilitation service type in Australia [1]. Most consumers referred to CCUs are experiencing severe and persisting mental illness (SPMI) and have complex care needs. At a CCU, consumers reside in co-located independent living units with 24-h staff support. The support is rehabilitation-focused, emphasising independent living skills development and community integration. These services emerged during the de-institutionalisation process to meet the needs of former long-stay psychiatric inpatients [9, 10]. While initially intended to provide a ‘home for life’, CCUs now provide time-limited (i.e., 'transitional') ‘recovery-oriented’ rehabilitation. Public mental health services in Australia are guided by ‘A national framework for recovery-oriented mental health services’ [11]; this emphasises the importance of personal recovery concepts and listening to the voices of lived experience. Consistent with this framework, many contemporary CCUs have reduced emphasis on clinical staffing. For example, under the ‘integrated staffing model’, peer support workers (PSWs) who have a lived experience of mental illness and recovery occupy most multidisciplinary team positions. The CCU model aligns with STAX-SA Type 2 services [12] (i.e., staff on-site, high support, strong emphasis on move on, congregate setting) available in the United Kingdom and several European countries [8, 13].

Transitional rehabilitation services aim to support a person's increased independence in the community post-discharge. Despite this, minimal research has examined contemporary consumers' post-discharge outcomes and experiences [13, 14]. Recent studies from Italy [15] and Finland [16] noted favourable post-discharge outcomes, and in the case of the Finish study, subjective improvements in recovery and quality of life (based on single item self-report scales). In the Australian context, a recent study found that most consumers experience reliable improvements in mental health and well-being and hospital utilisation (but not disability) when the year pre-admission and post-discharge is compared [14]. However, no research is available exploring the post-discharge experience from the consumer perspective. Early qualitative evaluations of the post-discharge experiences of consumers who had come to CCUs following long-stay inpatient care more than 20 years ago [9, 17], emphasised ongoing challenges in the community, including impoverished social networks and accommodation instability.

A mixed-methods longitudinal comparative evaluation of three CCUs commenced in Queensland, Australia, in 2014. Two of these units trialled the integrated staffing model, and the third operated the traditional clinical staffing model. The evaluation considered the impact of these staffing configurations on consumer experiences and outcomes. Findings to date have identified that consumers bring positive expectations to these services [18] and describe personal goals relevant to the service model [19]. However, accommodation rather than the opportunity for rehabilitation was the most frequently cited reason for choosing to come to a CCU [19]. 12–18 months after coming to the service, most consumers described the service positively and in a manner generally consistent with principles of recovery-oriented care [20]. Meaningful differences have not emerged between the integrated and clinical staffing model in the experience [20] and care outcomes (admission-to-discharge) [21]. However, consumers supported under the integrated model explicitly valued the availability of peer support workers.

It is unclear whether contemporary community-based residential rehabilitation consumers continue to experience the impoverished social lives and other challenges on discharge that were observed 20-years ago. This qualitative study aimed to explore consumers’ experiences twelve-to-eighteen months after leaving a CCU based on three questions: (1) what does life look like after leaving the CCU, (2) has the CCU impacted their life, and (3) how could the CCU experience be improved? The knowledge arising through this research was expected to guide an understanding of these services' real-world impact (or lack thereof) from the consumer perspective.

## Method

### Ethical clearance, study protocol and related data.

Ethics approval was provided by the MSAMHS Human Research Ethics Committee (HREC/14/QPAH/62); all participants provided voluntary written informed consent. The study design and reporting of findings were completed with consideration of the COREQ guidance [22] (see Supplementary Material). The full protocol for the mixed methods evaluation within which this study is nested provides a detailed description of the research team, analytic methods, and study context [23]. Descriptive statistics for participants involved in the interviews reflect a subset of the complete admission cohort data [24].

### Study context

CCUs are clinically operated public residential mental health facilities that provide transitional rehabilitation support to people affected by SPMI. These services aim to provide an enabling environment that facilitates positive risk-taking and encourages increased independence in the community. Accommodation is available in independent living units organised in a cluster-housing arrangement. Residents are expected to transition to an alternative residence in the community after 6-to-24 months. A range of therapeutic interventions are available on-site, including Cognitive Behaviour Therapy, Cognitive Remediation Therapy, and social cognitive interventions. The multidisciplinary team aims to provide recovery-oriented care by facilitating consumer goal setting, participation, and independence [25]. The three CCUs under investigation have a shared model of service and governance structure [26], and work with similar consumers [24]. One of the units opened in 2011 and uses the ‘clinical staffing model’, where nursing staff comprise the majority of the staff. The other two units opened in 2014/15 and operate an ‘integrated staffing model’, where Peer Support Workers comprise the majority of the staffing profile.

### Study team

All members of the study team, except for CM, have worked in the public mental health service where the study took place. FD, MP, RW, and SP are professionals from diverse disciplines, including lived experience (MP), medical (FD & SP), and social work (RW), who have directly worked in the CCU context. MC (psychologist) and MW (social worker) were employed in non-clinical research support roles within the broader hospital service. CM is a university-based academic with a background in anthropology who specialises in mixed methods translational research projects. The parent study was initiated by FD and led by SP, who shared a desire to understand the impact of different rehabilitation staffing configurations, the effectiveness of the service model and how it could be improved.

### Participants

Participants were drawn from a convenience sample of former residents who were discharged 12–18 months before their interview (data collection period 02–04/2019). Participation was prioritised based on the earliest consent and interview availability. No exclusion criteria were applied. Attempts were made to contact thirty-three consumers by telephone: 17 completed interviews either in person or by telephone, depending on their preference; 12 could not be contacted; and 4 declined to participate. The final sample approximated that in earlier work with this consumer group where thematic saturation was considered to be achieved [18, 20].

### Interview process

Semi-structured interviews were used to obtain rich information about the consumer experience [27]. The interview schedule (developed by SP, CM, and FD) focused on three key questions: (1) how have you been going since leaving the CCU, (2) how well did the CCU prepare you for living in the community, and (3) how could the CCU experience have been improved? A female independent research assistant (MC), who had no prior relationship with participants, completed all interviews and wrote field notes to assist with transcript intervention, except for the first two interviews being led by SP to orient her to the interview process. Audio-recording of the interviews were transcribed and de-identified by an independent transcription company.

After three interviews were completed, MC, SP, and CM reviewed the field notes to consider the adequacy of the interview schedule. The schedule was adapted to include optional prompts exploring therapeutic programs and how their current living situation compared to before and during their stay at the CCU. These prompts were not asked until the end of the interview and only used if this information was not spontaneously provided.

### Analysis

An inductive qualitative content analysis was completed by SP that followed the guidance of Erlingsson and Brysiewicz (2017); this was facilitated by the NVivo12 [28] software. All transcripts were read and then re-read in full to generate single-paragraph summaries of the main points of each interview. Then, the text of each transcript arising from the three interview questions was then divided into meaning units, which were condensed into codes, categories, and themes. A continuous reflective process was followed with meaning units continuously revised as the analysis progressed in consultation with the research team.

**Fig. 1:**
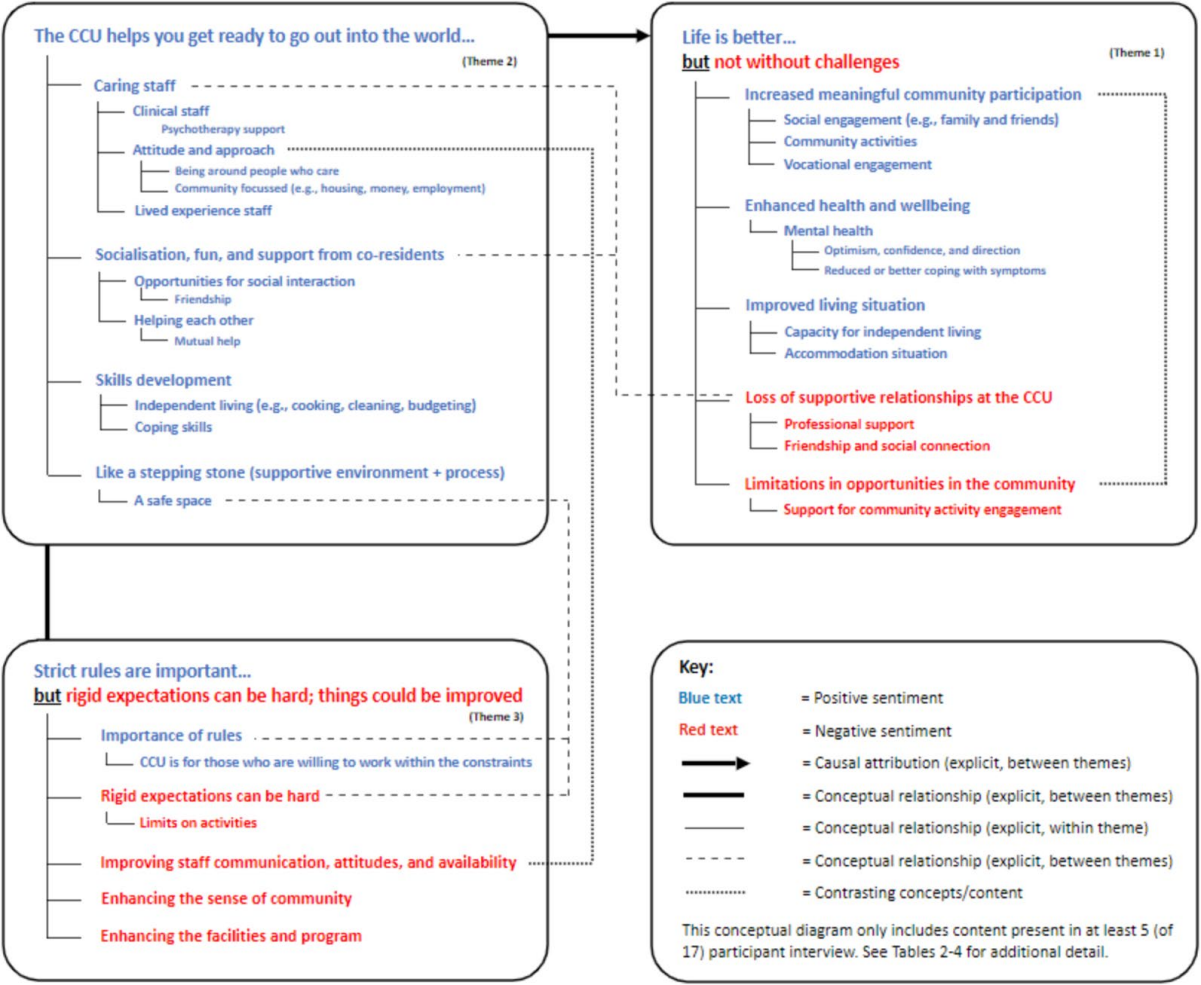
Consumer reflections 12–18 months after discharge from community-based residential mental health rehabilitation: Conceptual model of key themes, sub-themes, and associated content

The relationships between the key themes emerging across the dataset were depicted in a conceptual model, with emphasis placed on the explicit relationships between content areas made by participants rather than inferred by the researchers (the final version is provided in Fig. 1). The research team met to review the coding and conceptual model, and consider whether inductive thematic saturation [29] had been reached, and how adequately the themes were grounded in the data. Thematic saturation was indicated by the minimal emergence of new codes or themes from the later interviews.

In reporting the ‘Results’, transcript extracts best illustrating key themes were selected based on the agreement of all authors. MP provided feedback on the extracts and themes based on his relevant lived experience. Semi-quantifiers were used to indicate the proportion of participants associated with a given coding element [30]: few (n = 1–4/17); some (n = 5–8/17); half (n = 9/15); most (n = 10–16/17); and all (n = 17/17). Unique participant identifiers are included with illustrative transcript extracts designating the staffing model of the CCU site (CLIN = ‘clinical staffing model’, INT = ‘integrated staffing model’) and a unique three-digit random number for each participant (range 001–300, e.g. CLIN001). This approach is consistent with that taken in the parent mixed methods study's two consumer ‘expectations’ papers [18, 19] but differs from the consumer experience paper where ‘CON’ (i.e., ‘consumer’) was added between the staffing model and participant identifier.

## Results

Seventeen interviews were conducted (average duration 27.4 min, *SD* = 9.3). Participant characteristics are summarized in Table 1. Qualitative analysis identified themes that were generally consistent across participants from the clinical and integrated staffing model sites. Participants described their life as “better but not without challenges” 12 to 18 months after leaving the CCU (Theme 1, predominantly linked to responses to Question 1). They expressed the view that “the CCU helps you get ready to go out into the world” and that this experience had made a positive contribution to their life situation (Theme 2, predominantly linked to responses to Question 2). However, participants also acknowledged that their experience at the CCU was not always easy, stating that “strict rules are important but rigid expectations can be hard; things could be better” (Theme 3, predominantly linked to responses to Question 3). Figure 1 summarises the inter-relationships relationships between these themes as evident in participant transcripts.

**Table 1:** Participant characteristics

Staffing model	Clinical (n = 6)	Integrated (n = 11)	Total (N = 17)
Demographics (on admission)			
Mean age in years (SD)	28.2(5.1)	34.5(11.3)	32.24(9.8)
Unemployment ^a^	83.3%	90.9%	88.2%
Male sex	66.7%	72.7%	70.6%
Living with family pre-CCU	50.0%	81.8%	70.6%
Disability Support Pension	50.0%	63.6%	58.8%
Education level ≥ 12 years	66.7%	36.4%	47.1%
Australian born	16.7%	18.2%	17.6%
Treatment (on admission)			
Community-based referral	50%	54.5%	52.9%
Involuntary treatment order	66.7%	27.3%	41.2%
Guardianship order	16.7%	–	5.9%
Depot use	33.3%	45.5%	41.2%
Clozapine use	50%	36.4%	41.2%
Diagnosis (on admission)			
Primary diagnosis F20-29.x ^b^	66.7%	100%	88.2%
Comorbid nicotine dependence	16.7%	72.7%	52.9%
Comorbid substance use disorder ^c^	16.7%	36.4%	29.4%
Significant physical health issue ^d^	33.3%	27.3%	29.4%
Comorbid developmental disorder	16.7%	18.2%	17.6%
Comorbid PTSD/Trauma history	16.7%	–	5.9%
Comorbid OCD	16.7%	–	5.9%
Comorbid personality disorder	–	9.1%	5.9%
Discharge information			
Length of CCU stay (years)	1.33(0.37)	0.83(0.66)	1.01(0.62)
Planned discharge	83.3%	54.4%	64.7%
Involuntary treatment order	66.7%	27.3%	41.2%
Guardianship order	16.7%	-	5.9%
Accommodation			
Independent living	50.0%	45.5%	47.1%
Living with family	33.3%	36.4%	35.3%
Supported accommodation	16.7%	9.1%	11.8%
Acute inpatient care	16.7%	–	5.9%

^a^Any consumer not engaged in a paid or unpaid vocational activity, including volunteering

^b^ICD-10 classification including schizophrenia and schizoaffective disorder

^c^Excluding tobacco

^d^Score on HoNOS item 5a of 2 or above

### Life is better but not without its challenges (Theme 1)

All participants described improved quality of life compared to before they came to the CCU (see Table 2). Reported improvements included meaningful community participation, enhanced health and well-being, and more desirable living situations. Most participants described increased social connection (e.g., seeing family and friends) and improved mental health (e.g., optimism, confidence, reduced or better coping with symptoms).

*[Life is] good. I’ve got my own place… I cook for myself… I have more confidence cooking… and living on my own. I thank the CCU for that and the workers… [Buying food], I do that with a support worker… I thank CCU for helping me with that, and… trying to get me catching buses – I do that. I catch buses and trains.* [INT258]

*I look back and I realise quite a bit of it was delusional… just I went there with the understanding that I could spend some time in this introspective way figuring out what I was going through… [the CCU] promotes self-confidence and esteem.* [INT116]

**Table 2:** Life is better than before, but not without challenges (Theme 1): Sub-themes, categories, codes, and illustrative transcript extracts

Sub-theme	Category	Code	Illustrative transcript extract(s)
Increased meaningful community participation^e^	Social^e^	Seeing family^c,*^	I am seeing them a bit more [family]… [INT248]
			…I spend a lot more time just chatting and things. [INT116]
		Seeing friends^c^	[F]riendship wise… I've got some more people around me sometimes that know me. [CLIN141]
			I am meeting with friends more than I have in years…. [INT116]
		Meeting new people^b^	It's better… I have got my own car, I am independent, I'm paying bills and meeting new people. [INT158]
		Communication skills^b^	For a long time…I didn't understand how to socialise…. so the CCU was good to get going… [INT015]
	Community^c^	Spiritual participation^b^	I'm out and about a lot… we go witnessing with our church group. [CLIN125]
		Physical activity and recreation^b^	I'm doing Tai Chi with a whole heap of people… I've got my swimming hole. [INT015]
		Entertainment^b, ^^	[G]oing to the city and eating, shopping, things like that… [INT116]
		Services and supports^b^	Keep myself busy… go the drop-in centre… every day and have a coffee… [INT285]
	Vocational^c^	Looking for work^b^	I am really oriented in looking for a hard job… [CLIN141]
		Working^b^	In the kitchen… I work as an assistant… keeps me busy. [INT035]
Enhanced health and wellbeing^e^	Mental health^e^	Optimism, confidence, and direction^c^	[P]rior to going into the CCU I was wanting to end my life… I'm not like that now. [INT248]
			My mind state is more positive… I'm staying positive, I'm looking at the long-term goals… [CLIN041]
		Reduced/better coping with symptoms^c^	I don’t have delusions. I don’t even have voices anymore… [CLIN141]
			My beliefs are a bit more sane, before [and at the CCU] they didn’t really make sense… [INT234]
		Medications (positive)^b^	[Taking medication and attending appointments]… I don't want my life to fall apart again… [INT208]
	Physical fitness^b^	Increased physical fitness ^b^	I still go along to swimming on Thursday… I’ve actually lost weight since I’ve been at CCU… [CLIN141]
Improved living situation^e^	Capacity for independent living^c^	Cooking^b^	They prepared me well… the cooking skills. [CLIN136]
		Cleaning^b^	[CCU helped with] cooking, cleaning, relationships, getting accommodation… [INT035]
		Budgeting^b^	I'm just fully independent now, and I, I manage all the bills and stuff like that. [INT158]
		Transport access^b^	I catch buses and trains. I thank the CCU for the confidence for that. [INT258]
	Accommodation situation^c^	Living independently^b^	Beautiful… I get my own house… a one-bedroom unit accommodation. [INT155]
		Living with supportive family^b^	I’ve got a dream life. I just like wait for him [uncle] to finish and we go fishing or something… [INT015]
Ongoing struggles^e^	Loss of supportive relationships at the CCU^d^	Professional support^c^	Would be good to have… an occupational therapist… don't see anything moving forward… [INT248]
			CCU had more of an understanding from a medical perspective, they were much better. [CLIN041]
		Friendship and social connection^c^	I don't have the social support. I've got my… family, but I don't have the friends [at CCU]. [CLIN136]
			I miss the people, the workers and that at CCU. They were wonderful. [INT285]
	Limitations in opportunities in the community^d^	Support for community and activity engagement^c^	[C]ooked dinner together… had ping-pong matches together. I felt really supported there. [CLIN136]
			[Missing] swimming, the walks, even the bus trips, getting a Christmas present… [CLIN141]
		Financial constraint^b^	[T]here is an issue to do with financing it [access to desired instrument to play with friends, INT116]
		Unable to translate new skills^b^	They show you how to budget… [but] I'm not getting enough money in to be able to budget. [INT248]
			I didn’t use much of it… [living skills], my mum does most of that… I’m not living by myself. [INT035]
		Transitional accommodation^b^	I'm here temporary until I get my Housing Commission house. [INT155]

^a−g^:^a^(n = 1); ^b^(n = 2–4), ^c^(n = 5–8), ^d^(n = 9), ^e^(n = 10–13), ^f^(n = 14–16), ^g^(n = 17)

^*^:One participant described negative aspects of increased family interaction in the context of co-habitation

^^^:One participant described ongoing limitations in recreational engagement, but this not described as being a new issue

However, most participants also described ongoing struggles and limitations in opportunities in the community. Half the participants reflected on loss of the supportive relationships within and environment of the CCU (e.g., professional staff and co-resident friendship contact), and limitations in opportunities and supports to engage in desired activities.

*It's quite different [now] because I don't have the social support… I don't have the friends that I had as I [had] at CCU.* [CLIN136]

*I miss the people, the workers and that at CCU. They were wonderful… but it’s good to be independent in my own place.* [INT285]

### The CCU helps you get ready to go out into the world (Theme 2)

Participants emphasized the CCU acting as a ‘stepping stone’ towards being ready to go out into the world (i.e., the supportive people, processes, and environment working increasing one’s future independence and quality of life). Features of the CCU that were described as facilitating this were the availability of supportive and caring people (both staff and co-residents); also, the opportunities for social interaction and skills development (e.g., cooking, cleaning, and budgeting). Some participants emphasised the importance of the CCU providing a ‘safe space’ to work things out in their own way, as well as the contribution of the rules (e.g., prohibition of substance use and curfews) as supporting their sense of safety while living at the unit (see also *Theme 3*).

All participants described valuing the supportive actions of staff in the CCU environment. Most participants discussed valuing contributions of clinical staff (e.g., psychotherapy, enabling physical activity, and medication management). Furthermore, most participants from the integrated staffing model sites specifically emphasised the value of PSWs (e.g., availability and helpfulness, as role models, providing empathic understanding, and knowing the right way to interact). The caring attitudes of staff and their focus on the community (e.g., housing, finances, and employment) was viewed as an important contributor to positive changes occurring through the CCU experience (see also *Theme 3)*.

*[It] brought me back because you're around a lot of people that actually care about you. It made me feel really good and it pushed me to want to do good things, like better things with my life… [INT248]*

*[G]oing to the CCU just helped me – I’m growing even faster… it’s like a stepping stone, and all they had to offer was great... [INT158]*

*[T]hey improved on my medication taking, like how to take medication… [I]t made me be able to deal with crises that I'd have by myself… it helped me deal with things when I'm by myself - if I have a psychotic episode by myself, I'm able to deal with it. [CLIN136]*

### Strict rules are important but rigid expectations can be hard; things could be improved (Theme 3)

Half of the participants thought certain rules and expectations at the CCU were very strict, including those relating to participation, curfews and visitation, and substance use. Most of these participants acknowledged a need for these rules, particularly in relation to maintaining safety. However, a few expressed reservations about having to live under such structured conditions including its impact on their freedom to engage in desired activities (due to expectations of engagement and curfews) (Table 3). Some indicated they would only recommend a CCU to those people who were willing to work within such constraints. A few participants provided specific recommendations about how the CCU experience could have been improved through enhancing the sense of community (e.g., staff efforts to facilitate increased contact with family and friends), as well as the facilities and program (Table 4).

*I went in there thinking that I could stay there until I get really right in life and get all the lessons that I need… I wasn’t there long enough for it all to happen… [later recommending] maybe just if they could have a little more leniency… they’re pretty strict… you don’t have complete freedom… you have to go outside, even if it was raining, to have cigarettes… you weren’t allowed to go to the pub until a certain time on certain days…* [INT208]

*I noticed there was stronger points about my everyday living at CCU because it was more of a stricter place [than] I’ve ever been through*… *[I]t wouldn’t attract the right people if it wasn’t strict.* [CLIN141**]**

*[S]ome of the rules were quite strict but that’s kind of a good thing, like having the safety there…* [INT248]

**Table 3:** The CCU helps you get ready to go out into the world (Theme 2): Sub-themes, categories, codes, and illustrative transcript extracts

Sub-theme	Category	Code	Illustrative transcript extract(s)
Caring staff ^g^	Clinical staff ^^ f^	Psychotherapy support^*c^	[CRT] …It helped to work out the problem solving and how to deal with… everyday problem[s]. [INT155]
			When I first did CBT, it helped me to figure things out by going through them. [INT116]
		Physical activity support^b^	[Physical activity] made me feel really… energised, not flat and depressed and bored. [CLIN125]
		Medication^b^	I don’t have delusions. I don’t even have voices anymore… Just the right medication. [CLIN132]
	Attitude and approach ^e^	Being around people who care^c^	Made me feel really good and pushed me to want to do… better things with my life. [INT248]
			Knowing that people cared. That was the best thing. [INT033]
		Community-focussed (e.g., housing, money, employment)^c^	They helped me to connect, to [a community-based non-government organisation]. [INT155]
			I was happy… they gave me, support for, an apartment and all those kind of things. [CLIN141]
		Educating (increases understanding)^b^	[Valuing] their understanding of my…. mental/medical condition… [CLIN041]
			Getting other people’s perspectives on my problems with medication, schizophrenia… [INT035]
		Identifying what is needed and providing choices^b^	I was lost and confused in my mind; I wasn't sure of the direction where I want[ed] to take it. [T]hey gave all these options to support you in the future so you can have a good, positive experience… [CLIN041]
		Fostering hope ^b^	They taught me that I can really achieve… since then I've had the confidence to start saying… I want to get a job… get off the pension… I've started realising that I can do stuff for myself… [INT015]
	Lived experience staff ^c^	Helpful and available^b^	[They] lift you up… when you're feeling down… always there to listen when you need to talk. [INT248]
		Role models with shared experience^b^	[Realising] other people go through that. Other people hear voices… made me feel like… I'm not the only one here that has an issue… just to see them working… it was okay like; life can get better… [INT248]
		Empathic understanding^b^	Because they can understand they’re more compassionate. [INT285]
		Knowing the right way^b^	They could interact with us in the right way… most of them have gone through similar stuff. [INT234]
Socialisation, fun, and support from co-residents ^f^	Opportunities for social interaction ^c^	Friendship^c^	I had a lot of free time… to socialise with the people… making friendships, having fun times. [INT208]
			[CCU] got me involved with people… I gained a lot of friendship at CCU. [CLIN136]
		Shared activities^b^	I enjoyed the social aspect of it [the discussed regular trips to a restaurant with CCU peers]. [INT116]
	Helping each other ^c^	Mutual help^c^	Trying to help them at the same time as [them] helping you… [CLIN041]
			Going to CCU was better… Sharing your problems rather than carrying them on your own. [INT035]
		Benefits of other’s experience^b^	[Knowing] you're not alone… other people…have the same symptoms and things that you have. [INT248]
Skills development ^e^	Independent living skills ^e^	Cooking^e^	Participation in groups, cooking, keeping the house clean… just important in life to know. [INT208]
			Independently cooking and cleaning, to get that exposure, was really good for me. [INT035]
		Cleaning^c^	[Cooking and cleaning skills development]… Important so it can help me to get independent… [INT155]
			Cleaning, just how to live independently. [INT033]
		Budgeting^c^	I'm good at budgeting now… 'Cause if you don't budget… you become really unwell. [INT280]
			Budgeting [group]… I use it every day… I put money away every fortnight… [CLIN136]
		Medication management ^b^	They helped me how to get, they told me how to take the meds, without supervision. [INT115]
	Coping skills ^c^	Meditation, mindfulness, relaxation^b #^	Meditation was good… just clearing my mind and putting it all in a different place. [INT248]
			Mindfulness and the breathing techniques… it's very relaxing. [INT285]
		General^b^	They taught me how to deal with things and not rely on medication. [CLIN136]
	Communication ^b^	-	Teaching me how… to say no to people… [INT280]
Like a stepping- stone (supportive environment and process) ^d^	A safe space ^c^	Time and space to work things out your own way ^b^	[Allowing] time to heal… going somewhere where you have all these people around you, and you can, like, talk to them, and talk about your problems and stuff… [INT234]
		Supportive rules^b+^	Just having that feeling of safety… the rules and regulations… to be back at night like by nine o’clock if you go out. Certain things that they had like put in place. [INT248]
	Activities and opportunities ^b^	Structured activity^b^	They knew [what] was right for that structure… There was fitness, and then external things you could do…. Those sort of things were really good [because] it had a good structure to it. [CLIN041]

^**a−g**^Number of participants: ^a^ (n = 1/17); ^b^ (n = 2–4/17), ^c^ (n = 5–8/17), ^d^ (n = 9/17), ^e^ (n = 10–13/7), ^f^ (n = 14–16/17), ^g^ (n = 17/17)

^**^**^Participants also described the value of specific clinical disciplines: Dietician (n = 2), Medical (n = 2), Psychology (n = 1), Social Worker (n = 1)

^*^Predominantly focused on group-based psychotherapy support, specific interventions CBT for Psychosis (n = 3/17), Cognitive Remediation Training (CRT) (n = 3/17), Social Cognition and Interaction Training (SCIT), relapse prevention planning, sensory modulation (all n = 1, note one participant also described specifically finding SCIT unhelpful)

^~^Inclusive of staffing motivating, displaying respect, acknowledging your suffering, and communication with family

^#^One participant explicitly described not valuing participation in meditation, mindfulness, and relaxation practices at the CCU

^+^Three participants discussed negative aspects in residing in a supportive environment, with reference to the risks of dependence (n = 2/17) and self-consciousness limiting ability invite visitors (n = 1/17)

**Table 4:** Strict rules are important but rigid expectations can be hard; things could be improved (Theme 3): Sub-themes, categories, codes, and illustrative transcript extracts

Sub-theme	Category		Code	Illustrative transcript extract(s)
Strict rules are important but rigid expectations can be hard^d^	Importance of rules^d^		CCU is for those willing to work within the constraints^c^	[Strict rules…] it was very hard the way they were training us but… I appreciate how hard they pushed us. [CLIN141]
				It’s like a bit inhibiting, but in a positive way… [INT116]
			Keeping you safe^b^	Rules and regulations… [are] strict because of confliction between people… it’s about [maintaining a] cleanliness… [it] didn’t attract any bad people [CLIN141]
			Avoiding substance use^b^	Anyone going through withdrawals or… additions, it helps… To be away from… the temptation. [INT285]
	Rigid expectations are hard^c^	Limits on activities^c^	Movement^b^	The CCU is like you have to be on time… I go out on the weekends… I stay home because I’ve things to do at home [not because other expect it]. [INT258]
			Visitors^b^	It’s good to be independent… you can have visitors whenever you want [now]… [INT285]
			Prohibitions^a^	Opportunities come and go [e.g., jury duty]… doing that would’ve been a good approach to recovery… they wouldn’t let you do a lot of things. [CLIN132]
		Being forced^b^	-	It was like if you want to stay here you’ve got to do stuff… I was a bit forced. [INT208]
		Substance use^b^	Need for leniency^b^	If they had a little bit more leniency… three chances… then that’s it, you’re out… [INT208]
			Favouritism (inconsistency)^a^	[Three were smoking] weed… [the boss] kick[ed] out two… it's called favouritism. [INT258]
		Timeframes^a^	Not long enough^a,^^	I went in there thinking that I could stay there until I get really right with life… I wasn't there long enough for it all to happen… [INT208]
		Independence^a^	Staff not sharing milk^a^	The boss… [doesn’t let staff] share… milk goes off they were chucking the [it] out… [INT258]
			Purchasing own medication^a^	[You] have to pay for it [depot] but [now I]… go straight to mental health to get it… [INT258]
Improving staff communication, attitudes, and availability^c^	Communication^b^		Transparency (e.g., staff, care timeframes, housing outcomes)^b^	[I] wish we knew more about where the staff came from… [Also] they sometimes say… ‘we’ll look after you for two years’, but they only [get] eight months… [INT015]
			Clearer communication (rules)^a^	They could have explained it a bit more to be honest… [INT208]
			Family involvement^a^	They could have contacted [my] grandparents [coordinating child visits] [INT248]
	Attitudes^b^		Increase strengths emphasis^b^	Trying to find each] person’s true strength, and then from there trying to relate that strength to how it can help him with his negative side… [CLIN041]
			Being less interrogating^a^	They’re so interrogating… I wish they could be more comfortable… [CLIN141]
	Availability^b^		Providing more assistance^b^	To get a house with Housing Commission… [and] money from Public Trust… [INT155]
			Medical continuity^a^	I think I saw three different [Registrars] while I was there. It just makes it harder because you have to repeat yourself and you get different—different diagnoses, conclusions, opinions…
Enhancing the sense of community ^c^	Co-residents^b^		Gender imbalance^a^	Sometimes I wished there was [another] girl there. [INT234]
			Limiting substance use^a^	Too many drugs… it was just a temptation… [I]t was just not good to see… [INT033]
			Over-involvement^a^	Patients stick their nose in it… quite often I felt I was ganged up on… [INT258]
	Family and friends^b^		Facilitating family contact^b^	I had my parents around you know maybe twice but that was all you know? [CLIN132]
				[To have] my children visit me. [INT248]
			Maintaining contact with friends^b^	Conscious that people… might have impressions about what… type of place it was… [CLIN132]
Enhancing the facilities and program ^c^	Therapy and recreational program^c^		Increased art and craft^b^	I could probably, like, think about a bit more art. [INT234]
			Less emphasis on computers^a,*^	Courses were done by computer, I don’t know how to work a computer. [INT280]
			Enhance meditation program^a^	The meditation class, I thought it was a bit bogus… [INT116]
			Massage availability ^a^	It would be good if they incorporated massages… [INT248]
	Facilities^b^		Less institutional^a^	If the unit seemed more like… private rental[s] rather [than]… part of a little thing… [CLIN132]
			Unit cleanliness (on arrival)^a^	The unit was very dirty when I first moved in… wasn’t cleaned properly. [INT035]

^**a−g**^Number of participants: ^a^(n = 1/17); ^b^(n = 2–4/17), ^c^(n = 5–8/17), ^d^(n = 9/17), ^e^(n = 10–13/7), ^f^(n = 14–16/17), ^g^(n = 17/17)

^**^**^Another participant expressed the contrasting review that more time limits on the duration of stay were relevant to reduce the risk of dependency [INT208]

^*^The Cognitive Remediation therapy program was computer based

## Discussion

Consumers generally reflected positively on their life experiences over the twelve-to-eighteen months following discharge from a community-based residential mental health rehabilitation unit. They described improvements in their levels of community participation, health and wellbeing, and living situation (Theme 1). The CCU was described as having an instrumental role in supporting their readiness to go out into the world through the support provided by staff and co-residents, and opportunities for living skills development (Theme (2). While sentiment about the CCU was generally positive, most consumers acknowledged both the value and challenges of residing under ‘strict rules’ and rigid expectations (Theme (3). Most consumers also described experiencing ongoing struggles in the community, including loss of professional and social supports experienced in the CCU environment, and limitations in opportunities in the community. Meaningful differences between participants supported under the clinical and integrated staffing models did not emerge; however, most participants who were supported under the integrated model described specifically valuing the availability of PSWs.

### Considering the findings in the context of the broader literature

Our findings provide a stark contrast to post-discharge experience reported in the only other qualitative study exploring Australian CCU consumers’ post-discharge experiences. Chopra et al. interviewed consumers who had been part of the original 1996 de-institutionalisation cohort of a Victorian CCU eight years following their admission (median duration post discharge ~ 3-years). Their study identified high levels of unmet needs and impoverishment. A pessimistic interpretation would be that the positive experiences reported in our study reflect the shorter follow-up duration; it is possible that the gains made following residential rehabilitation care may be lost over time. Alternatively, the difference may relate to changes over the last twenty years in a broad range of factors, including available treatments, models of care, and the characteristics of consumers admitted to CCUs [1]; or factors unrelated to the CCU, such as stigma and social inclusion. Contemporary CCUs explicitly focus on the transitional nature of residential support and work with consumers who are less likely to have a history of institutionalisation. Our findings suggest the possibility that these rehabilitation services now support consumers in a manner more likely to result in sustained benefits.

The positive reflections on the impacts of the residential rehabilitation experience on post-discharge adjustment (Theme 2) mirror the limited subjective data reported in a contemporary Finnish study [16]. In that study, improvements were noted in subjective recovery and quality of life, with most consumers indicating that the indicating that the rehabilitation experience had positively impacted their lives (87.5%). Similarly, the subjective report of consumers in the present study were generally consistent with the positive findings from the limited quantitative studies considering outcomes post-discharge from contemporary community based residential rehabilitation services [14–16]. However, the description of enhanced community functioning by all participants (e.g., capacity for independent living) in our sample (Theme 1) contrasts with the failure to observe gains in clinician-rated disability (as measured by the Life Skills Profile-16) in an Australian sample where the 12-months pre-admission and post-discharge from a CCU were compared (2005–2014 admission data) [14]. An explanation for the difference between clinician-rated disability and consumer-experiences of function may be the known poor correlation between the LSP-16 and consumer-rated recovery measures [31].

### Alignment between consumer reflections, and consumer expectations and experiences

Earlier qualitative research conducted across the three study sites explored the consumers' expectations of a CCU (on commencement) [18, 19] and their experiences twelve-to-eighteen months after admission [20]. Reflections on the CCU experience in the present study aligned with what consumers had hoped to receive through the service, including personal transformation being achieved through living and engaging in a supportive transitional space [19]. Similarly, the focus on the importance of social relationships with staff and co-residents as being critical to the CCU described in the consumer experience interviews [18] was mirrored in their reflections. In the experience interviews some consumers described anxiety about transition, with fears about the loss of the CCU environment and support. A similar proportion of participants in the present study described a sense of loss associated with moving out of the CCU environment. Despite this, all participants in the present study provided positive reflections on their lives post CCU.

### Limitations

This research is exploratory and transferring the findings to other contexts would require careful consideration of the similarity of the consumers, services, and other features. Additionally, very few participants were involved in the earlier expectations and experience interview phases (n = 2/17). As such, conclusions about consumer perspectives over time is based on the assumptions that the findings from earlier phases were generalisable. Additionally, the small number of participants from the clinical staffing model site means it is possible that additional themes distinguishing the two staffing model approaches may have emerged if further interviews were conducted [32]. Furthermore, it is possible that consumers with more positive post-discharge experiences were more likely to participate in interviews.

A cross-sectional approach was taken to explore the post-discharge experience. While this permitted an understanding of how participants were going after leaving the CCUs, it did not enable a rich understanding of the process of recovery following the transition. For example, the interview schedule provided limited scope to explore important issues such as ongoing efforts towards occupational recovery [33] and the steps people took towards enhancing their everyday activities and community and vocational engagement. Future research focusing on these issues and using multiple interviews with individuals during the transition period may provide valuable guidance to support people leaving a CCU in the transition to independent community living.

The primary interviewer (MC) was employed by the health service operating the CCUs and was concurrently completing a clinically focused post-graduate program (psychology). Furthermore, the initial two interviews were led by SP, a psychiatrist who had worked previously at all three of the services under investigation. Another research team member (FD) was a senior clinician with clinical governance responsibilities across the three sites. The dual roles of these researchers brought sensitisation to relevant concepts and potential bias towards more positive evaluations and a clinical rehabilitation lens. Efforts to ensure the trustworthiness of the analysis included: initial coding being completed by a non-clinician who was independent of the sites under investigation (MC), involvement of an independent non-clinician researcher (CM), and a researcher with relevant lived experience (MP) in reviewing the adequacy of the coding framework.

## Conclusion

Consumers reflected positively on their lives post-discharge from a community-based residential rehabilitation unit and viewed the service as having supported improvements in their lives. While they acknowledged that the expectations and restraints associated with a transitional rehabilitation setting were challenging, these aspects of the care environment were generally viewed as necessary to support safety and engagement. The findings support an optimistic view of the appropriateness of community-based transitional rehabilitation translating into improved quality of life and independence in the community. This optimistic view contrasts the concerns about impoverishment and unmet needs emphasised in the only study exploring the post-discharge experiences of CCU consumers who had a prior history of long-term institutional care approximately two decades ago. The positive sentiment expressed by consumers about the value of a transitional residential rehabilitation environment suggests the need for caution in policy advocacy to abandon residential treatment in favour of exclusively funding permanent supportive housing solutions (e.g., Housing First). Our findings suggest that for some people living with mental illnesses, these services are valued and viewed as an enabling step to increased independence.

### Relevance for clinical practice

Residents emphasise the importance of their relationships with staff and others at a residential rehabilitation unit. Additionally, they acknowledge the important role of staff in maintaining a social environment that is safe and supportive, including making ‘tough but fair’ decisions that may include asking residents to leave when the rules are broken. While the demands of transitional residential rehabilitation can be challenging for residents, the outcomes achieved through engagement with this support tend to be highly valued. These findings should give clinicians confidence that their work is valued and consumers view it as leading to meaningful change in their life situation. Sharing this knowledge with consumers at their services may have relevance in instilling hope in the expectation of recovery.

## Supplementary Information

Below is the link to the electronic supplementary material.

Supplementary file1 (PDF 438 KB)

## Data Availability

The release of data associated with this project would require approval from the relevant Human Research Ethics Committee.
